# Assembly of PbTe/Pb-based nanocomposite and photoelectric property

**DOI:** 10.1186/1556-276X-8-191

**Published:** 2013-04-24

**Authors:** Zhaocun Zong, Hongxia Wang, Lingmin Kong

**Affiliations:** 1 College of Mathematics, Physics and Information Science, Zhejiang Ocean University, Zhoushan 316000, People's Republic of China

**Keywords:** Nanocomposite, Nanostructure, Arrays, Photoelectric performance

## Abstract

PbTe/Pb-based nanocomposite was assembled by combining the regular PbTe/Pb nanostructure and the Zn_*x*_Mn_1−*x*_S nanoparticles; the photoelectric property of the nanocomposite was measured *in situ*. The results showed that the through current of the nanocomposite had an obvious increase compared to that of the individual PbTe/Pb nanomaterial under the same irradiation conditions. The improvement of photoelectric performance would be attributed to the synergistic effect brought by the incident light and exciting light of the Zn_*x*_Mn_1−*x*_S nanoparticles. The result implied that the underlying mechanism could be used to improve the performance of nano-optoelectronic devices and the light-use efficiency of solar devices.

## Background

With the development of nanotechnology, complex micro/nanodevice assembly would gradually be a reality in the future. The various explorations in the aspects of nanomaterial preparation and performance at present provide the base for nano-engineering, in which the controllable preparation and unique performance of nanomaterials have been the keys of exploration. With the aim of exploiting new coupling phenomena and potential applications, nanocomposites have attracted much attention over the past decade [1-5]. The typical preparation way is through an *in situ* fabrication; the different components are integrated together to form a nanocomposite at the same time. For example, metallic nanocrystals could be incorporated into one-dimensional (1D) carbons to form a metal-carbon nanocomposite via an organometallic precursor-controlled thermolysis approach. Unprecedented physical and chemical properties become available due to the effects of spatial confinement and synergetic electronic interactions between metallic and carbonaceous components [6]. This type of nanocomposite has shown unique properties in some aspects including magnetic, catalytic, electronic, and thermoelectric properties [7-10]. Another preparation way is the surface recombination of several different individual nanomaterials using a physical or chemical method. Due to the complexity and importance of the nanomaterial surface property, this type of nanocomposite can more easily show the new phenomenon and unique performance. A semiconductor quantum dot such as CdSe was coated onto the surface of a silver nanowire. The emission energy of the excited CdSe quantum dot near the silver nanowire could couple to guided surface plasmons in the nanowire [1]. Especially, in the optical properties, this type of nanocomposite has attracted great scientific interest [11]. It is just the complexity of the interaction; different factors, including composition, size, and geometry of the nanostructures; and the distance between nanostructures that provide the challenge for quantifiable research and the mechanism achievement of a new phenomenon [12]. So, the preparation and synthesis of uniform nanomaterials in terms of morphology and structure provide the important precondition for the further study of material properties.

As a narrow bandgap semiconductor (approximately 0.32 eV, at 300 K), lead telluride (PbTe) has been extensively studied and used in optical detectors [13], laser devices [14,15], and thermoelectrics [16,17]. Compared to other semiconductor materials, low-dimensional PbTe semiconductors could more easily show the obvious quantum size effect on larger scales because of the larger Bohr exciton radius (approximately 46 nm). So, 1D PbTe nanomaterials have attracted intense scientific attention in recent years and have been synthesized by a variety of physical and chemical techniques [16-22]. The solution-based chemical synthesis and chemical vapor deposition have been usually utilized to synthesize single-crystalline PbTe nanowires, and the conventional electrical property measurement of PbTe nanowires has been achieved [16,23]. However, less attention has been paid to the preparation and unique property study of 1D PbTe-based nanocomposites at present.

In this paper, we first electrodeposited the nanostructure arrays made of a uniform PbTe/Pb nanostructure in size and composition. Then, the regular PbTe/Pb nanostructure arrays and the synthesized Zn_*x*_Mn_1−*x*_S nanoparticles were assembled to construct a PbTe-based nanocomposite. The photoelectric property measurements of the material were also performed *in situ* along with the assembly process of the nanocomposite. The measurement results showed that the photoelectric performance of the PbTe/Pb-based nanocomposite had an obvious improvement compared to that of the individual PbTe/Pb nanomaterial. The improved performance of the nanocomposite could originate from the synergistic effect brought by the incident light and exciting light of the nanoparticles. The underlying mechanism shows that the light-use efficiency (LUE) of the PbTe/Pb-based nanocomposite had an obvious increase compared to that of the PbTe/Pb nanomaterial.

## Methods

### Synthesis of nanostructure arrays by electrodeposition

**Figure 1:**
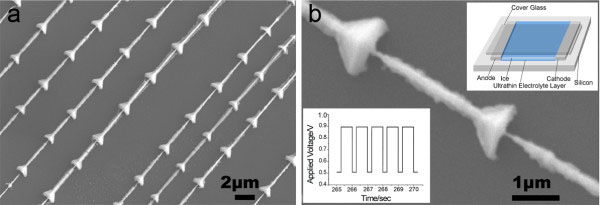
**Scanning electron microscopy image of the PbTe/Pb nanostructure.** (**a**) The representative SEM image of PbTe/Pb nanostructure arrays with a field of view of 30 μm (*w*) × 20 μm (*h*). (**b**) The SEM image of the single PbTe/Pb nanostructure. The upper right insert figure gives the central configuration schematic of the electrochemical cell. The lower left insert figure gives the applied voltage waveform. The applied voltage varies from 0.5 to 0.9 V in a square waveform with 1 Hz frequency.

In our experiments, the regular PbTe/Pb nanostructure arrays were electrodeposited on a silicon (110) wafer. The electrodeposition process was achieved in a few hundred-nanometer- thick electrolyte layer, which was controlled with an ultrathin electrochemical deposition setup [24]. The ion concentration in the ultrathin electrolyte layer had a key role to the initial morphology of electrodeposition, which attributed to the amount of crystal nuclei on the electrode at the beginning of the electrodeposition process. Owing to the deoxidized plentiful crystal nuclei, the higher ion concentration facilitated the form of a two-dimensional thin film at a lower potential in the electrolyte. When the ion concentration was lower, the amount of deoxidized crystal nuclei did not afford the needs of thin film growth, and the two-dimensional growth form would be replaced by the one-dimensional growth form. The schematic diagrams of the experimental setup were shown in Figure 1b.

The electrodeposition of the PbTe/Pb nanostructure arrays was carried out by applying a square wave potential with a frequency of 1 Hz (in Figure 1b) across the ultrathin layer. The electrolyte was prepared using analytical reagent Pb(NO_3_)_2_, TeO_2_ (Fluka, Sigma-Aldrich Corporation, St. Louis, MO, USA), and Millipore water (Millipore Co., Billerica, MA, USA). The ion concentrations of Pb^2+^ and HTeO_2_^+^ in the electrolyte were 0.005 and 0.001 M, respectively. The pH value of the electrolyte was adjusted to 1.87 by nitric acid. The treated silicon substrate (20 × 20 mm^2^) (Fluka) was first placed on the Peltier element. Silicon was treated using chemical erosion and oxidation process, which would bring an insulation and uniform thickness of the SiO_2_ layer on the surface of the silicon wafer. Next, the two parallel lead foil electrodes with 30-μm thickness (Fluka) were placed on the substrate and filled with the electrolyte. A cover glass was put on the electrodes, and the simple electrolytic cell was assembled. After that, the temperature control system consisted of a circulating water bath, and the Peltier element was used to solidify the electrolyte. Due to the partitioning effect, the solute in the electrolyte could be partially expelled from the solid in the solidification process. The concentrated electrolyte layer with 300-nm thickness was formed between the ice from the electrolyte and the SiO_2_/Si substrate when the temperature dropped to −5.20°C. The temperature played an important role to the control of the electrolyte layer thickness and concentration. The lower temperature could cause the solute in the electrolyte layer to be further expelled from the solid, which made the concentration of the electrolyte layer more concentrated. Meanwhile, the thickness of ice increased, and the electrolyte layer thickness reduced*.* The electrodeposition process was achieved by applying a square wave potential with a frequency of 1 Hz.

### Characterization techniques

**Figure 2:**
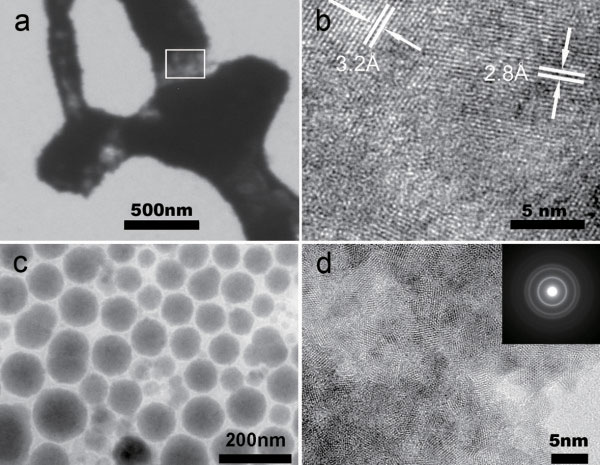
**The transmission electron microscopy characterization.** (**a**) The image of the electrodeposit shows the location where high-resolution TEM was performed. (**b**) High-resolution TEM image at the frame area of image (**a**) shows two groups of lattice fringes, corresponding to the PbTe(200) and Pb(111) lattice planes. (**c**) The representative morphology of Zn_1−*x*_Mn_*x*_S nanoparticles. The particle diameter is approximately 100 nm. (**d**) The high-resolution TEM image of the Zn_1−*x*_Mn_*x*_S nanoparticles. The inset gives the electron diffraction powder pattern of the sample.

The morphologies of the samples were characterized using field emission scanning electron microscopy (SEM; JEOL JSM-6700 F, JEOL Ltd., Tokyo, Japan) and transmission electron microscopy (TEM: JEM 2010 F, JEOL Ltd.), respectively. The controllable PbTe/Pb nanostructure arrays were shown in Figure 1a. The PbTe/Pb nanostructure material had a periodically changed morphology, and the length of the ordered arrays could reach a few hundred microns. The diameter of the single PbTe/Pb nanostructure changed from 100 nm to 1 μm, as seen in Figure 1b. The high-resolution transmission electron microscopy (HRTEM) image showed that there were two kinds of *l* grains at the location of the PbTe/Pb nanostructure, Pb and PbTe, as seen in Figure 2b. According to the basic electrodeposition theory, the different ions correspond to the different reduction potentials in the process of electrodeposition. In the preparation of the PbTe/Pb nanostructure, when the applied voltage was lower, only Pb^2+^ cations could be deoxidized; after the applied voltage became 0.9 V from 0.5 V, both HTeO_2_^+^ and Pb^2+^ cations were deoxidized together. Thus, the component of the nanostructure at the thin location was composed of PbTe grains and metal Pb. Figure 2c showed the representative morphology of Zn_1−*x*_Mn_*x*_S nanoparticles synthesized by the gas-liquid interface method [25], and the range of nanoparticle diameters was from about 50 to 150 nm. The HRTEM image showed that nanoparticles were made up of a lot of nanocrystals, as seen in Figure 2d.

## Results and discussion

### Simulation analysis of electric field vector distributions

In the preparation of the regular PbTe/Pb nanostructure arrays, the limitation of the electrodeposition room was a key factor. The preparation of one-dimensional nanomaterials could be achieved in the quasi-two-dimensional room by the reasonable control of electrolyte concentration and reduction potentials. Every PbTe/Pb nanostructure was composed of periodic growth parts with changed diameter. The controllable morphology mainly originated from two factors: one was the balance between the supply and the consumption of cations in the front area of the growth tip, while the other important factor was the applied voltage.

**Figure 3:**
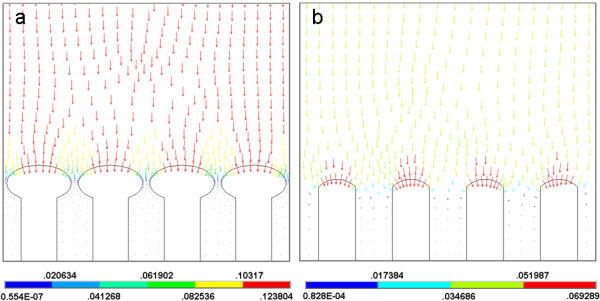
**The computed distributions of the electric field vector near the tips of the nanostructure arrays.** (**a**) The electric field vector distributions when the applied voltage became 0.9 V from 0.5 V. (**b**) The electric field vector distributions when the applied voltage became 0.5 V from 0.9 V.

The applied voltage varied from 0.5 to 0.9 V in a square waveform with 1 Hz frequency. In the electrodeposition process, there was a balance between the ion supply and ion consumption, which decided the range of nucleation regions at the growth tip. The potential determined the ion consumption; meanwhile, it also led the ion supply in the electrolyte. When the applied voltage was changed to 0.9 V, the previous balance between the supply of cations and the consumption of cations in the front area of the growth tip was broken. The increased potential would quicken the reduction rate of cations and change the distribution of electrical field at the tip of the nanowire. Once the electromigration did not provide enough ions for the consumption, the nucleation regions would shrink. Figure 3a showed the distribution of the computed electric field vector near the tips of the nanowire array model at 0.9 V. The computed results indicated that the electric field would become concentrated at the forehead of the whole growth tip. The distribution of electric field was uniform in the whole arrays and would make the nucleation regions shrink at every growth tip of the arrays. The distribution of electric field intensity would decide the locations of cations arriving in the electrolyte. Generally, the nucleation would not occur until the number of cations reached a certain amount. According to the distribution of the computed electric field vector at 0.9 V, the intense region of the electric field was from about 0.08 to 0.12 at the growth tip. Comparing the SEM image of the nanostructures and the distribution of the computed electric field vector, the suitable field intensity range of the nucleation regions should be from 0.082536 to 0.123804. So, the diameter of the followed growth part became thin. When the applied voltage was changed to 0.5 V from 0.9 V, the distribution of the computed electric field vector near the tips of the nanowire array model was shown in Figure 3b. The migrating ions would be redistributed at the different locations of the nanowire tip according to the distribution of electric field at the tip of the nanowire. According to the same electric field intensity span range of the nucleation regions, the electric field intensity range of the nucleation regions at the growth tip should be from about 0.069289 to 0.017384 at 0.5 V. The range in Figure 3b showed that the nucleation regions had extended to both sides of the tip from the growth tip when the applied voltage was changed to 0.5 V from 0.9 V. The migrating ions could first arrive at the region and start to be deoxidized. The lateral lower electric field intensity regions at the growth tip would not nucleate because of the shortage of cations. So, the diameter of the followed growth part would become wider gradually. The computed results exactly simulated the distribution of electric field intensity at the tip of the nanomaterials and coincided with the actual growth conditions of the nanomaterials.

### *In situ* assembly and photoelectric property measurement

**Figure 4:**
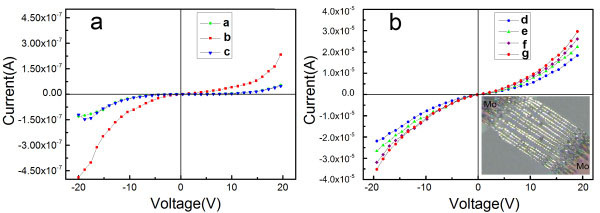
**The photoelectric performance measurement.** (**a**) The current-voltage characteristics of the single PbTe/Pb nanostructure before and after laser irradiation at 300 K *a* Without light irradiation; *b* under the 532-nm wavelength, 1 × 10^−3^ W/cm^2^ laser irradiation; and *c* restoration without light irradiation again. (**b**) The current-voltage characteristics of PbTe/Pb nanostructure arrays before and after assembling the Zn_*x*_Mn_1−*x*_S nanoparticles at 300 K. The lower right insert figure gives the optical micrograph of the PbTe/Pb array device with molybdenum electrodes. *d* Without light irradiation; *e* under the 532-nm wavelength, 1 × 10^−3^ W/cm^2^ laser irradiation; *f* combined a spot of Zn_*x*_Mn_1−*x*_S nanoparticles under the 532-nm wavelength, 1 × 10^−3^ W/cm^2^ laser irradiation; and *g* combined sufficient Zn_*x*_Mn_1−*x*_S nanoparticles under the 532-nm wavelength, 1 × 10^−3^ W/cm^2^ laser irradiation.

**Figure 5:**
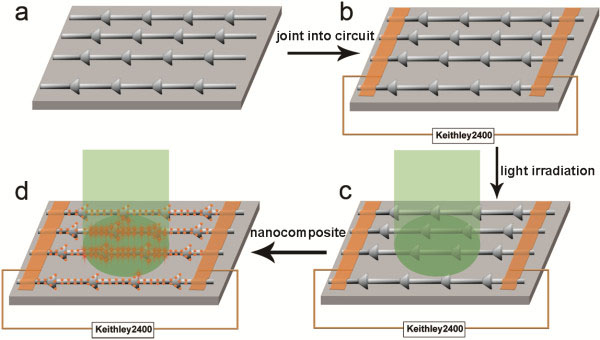
**The Schematic illustration of PbTe/Pb-based nanocomposite situ assembly and photoelectric measurement process.** (**a**) The electrodeposited PbTe/Pb nanostructure arrays on a substrate. (**b**) The circuit connection of PbTe/Pb nanostructure and its electrical performance measurement. (**c**) The photoelectric performance measurement of individual PbTe/Pb nanostructure. (**d**) The situ assembly of the PbTe/Pb-based nanocomposite and its photoelectric performance measurement.

The electrodeposited regular PbTe/Pb nanostructure is first jointed into the circuit by using e-beam evaporation, as seen in Figure 4b. The excellent conductive metal molybdenum is used as the electrode material*.* Then, the ethanol turbid liquid containing Zn_*x*_Mn_1−*x*_S nanoparticles doped with 1.26 mol% of Mn^2+^ content is gradually dripped into the PbTe/Pb nanostructure arrays. With the evaporation of ethanol, the capillary force drives the spherical nanoparticle to flow toward the PbTe/Pb nanostructure surface; finally, the Zn_*x*_Mn_1−*x*_S nanoparticle is deposited on the surface [26]. Comparing the changes of current versus voltage (*I*-*V*) curves before and after assembling the Zn_*x*_Mn_1−*x*_S nanoparticles, we study their photoelectric property under the 532-nm wavelength and 1 × 10^−3^ W/cm^2^ laser irradiation conditions. Figure 5 shows the schematic illustration of the *in situ* construction and photoelectric measurement process.

**Figure 6:**
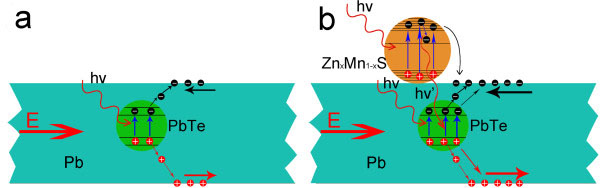
**The photoelectric mechanism schematic diagram.** (**a**) The carrier generation mechanism schematic diagram in the PbTe/Pb nanostructure under light irradiation. (**b**) The carrier generation mechanism schematic diagram in the PbTe/Pb-based nanocomposite under light irradiation.

The electrical measurements are performed by an ultrahigh vacuum system (1 × 10^−9^ Torr) at 300 K. All of the *I*-*V* characteristics under a high bias voltage are nonlinear, as shown in Figure 4. Figure 4a gives the *I*-*V* curves of the individual PbTe/Pb nanostructure before and after light irradiation. It is obvious that there is an increase of through current to the individual PbTe/Pb nanostructure device under the irradiation of a 532-nm wavelength laser. When the irradiation is stopped, the *I*-*V* characteristics of the device can be restored completely. HRTEM (Figure 2) has shown that the PbTe/Pb nanostructure is composed of semiconductor PbTe grains and metal Pb. In general, semiconductor grains embedded in the metal could effectively increase the resistance because of the scattering action due to the crystal boundary potential barrier. As PbTe is a narrow bandgap semiconductor, when the PbTe/Pb nanostructure was irradiated by the 532-nm wavelength laser, light irradiation could not only reduce the height of the crystal boundary potential barrier in PbTe/Pb nanostructure, but also generate more carriers. Figure 6a shows the carrier generation mechanism schematic diagram in the PbTe/Pb nanostructure under light irradiation. The two factors could result in the increase of PbTe/Pb nanostructure conductivity. The *I*-*V* curves of the PbTe/Pb nanostructure arrays before and after assembling the Zn_*x*_Mn_1−*x*_S nanoparticles are shown in Figure 4b. The *I*-*V* curves indicate that the assembly of the Zn_*x*_Mn_1−*x*_S nanoparticles further increases the through current under the same laser irradiation. The performance of the PbTe/Pb-based nanocomposite had an obvious increase compared to that of the individual PbTe/Pb nanomaterial. When the PbTe/Pb-based nanocomposite is irradiated by the 532-nm wavelength laser, the nanoparticles coated on the surface could be excited. The electron that absorbed photon energy would first jump to the conduction band from the valence band in the Zn_*x*_Mn_1−*x*_S nanoparticles. Due to the differences in the work functions of materials, the carriers would transfer between the two mutual contact materials. For the two materials constituting the PbTe/Pb-based nanocomposite, the electron would transfer from the Fermi-level higher Zn_*x*_Mn_1−*x*_S nanoparticle surface to the Fermi-level lower PbTe/Pb nanostructure surface, which would increase the carrier amount of the nanocomposite. In addition, the Zn_*x*_Mn_1−*x*_S nanoparticle is an important dilute magnetic semiconductor, and its bandgap can be adjusted by the doped contents of Mn^2+^ ions; the doping of Mn^2+^ ions brings the different electronic energy levels for Zn_*x*_Mn_1−*x*_S nanoparticles. When the excited electrons in the high energy level jump to the low energy level, the excess energy would be released in the form of photons. These released photons, together with the photons from the laser, would excite the PbTe grains in the PbTe/Pb nanostructure, so the excited carrier amount in the PbTe/Pb-based nanocomposite is more than that in the PbTe/Pb nanostructure. The detailed carrier generation mechanism schematic diagram in the PbTe/Pb-based nanocomposite is shown in Figure 6b. Therefore, the same light irradiation conditions make the PbTe-based nanocomposite generate more carriers; the through current in the PbTe-based nanocomposite can be further increased compared to that in the single PbTe/Pb nanostructure arrays under the same light irradiation. The underlying mechanism shows that the LUE of the PbTe/Pb-based nanocomposite had an obvious increase compared to that of the individual PbTe/Pb nanomaterial.

## Conclusions

In summary, the PbTe/Pb-based nanocomposite is assembled by combining the PbTe/Pb nanostructure arrays and the Zn_*x*_Mn_1−*x*_S nanoparticles. The photoelectric measurement shows that the photoelectric performance of the PbTe/Pb-based nanocomposite had an obvious improvement compared to that of the individual PbTe/Pb nanomaterial. The improvement of photoelectric performance could originate from the synergistic effect of the incident light of the laser and the stimulated radiation of the Zn_*x*_Mn_1−*x*_S nanoparticles on the surface of the PbTe/Pb nanostructure. The result implies that the underlying mechanism may be used to improve the performance of nano-optoelectronic devices and explore the novel properties of nanocomposites.

## Competing interests

The authors declare that they have no competing interests.

## Authors’ contributions

ZCZ carried out the material preparation, characterization and simulation analysis. HXW participated in the design and mechanism analysis of this study and drafted the manuscript. LMK carried out the photoelectric property measurement of materials. All authors read and approved the final manuscript.
